# Quantifying the Shape of Aging

**DOI:** 10.1371/journal.pone.0119163

**Published:** 2015-03-24

**Authors:** Tomasz F. Wrycza, Trifon I. Missov, Annette Baudisch

**Affiliations:** 1 Max Planck Institute for Demographic Research, Rostock, Germany; 2 Institute of Sociology and Demography, University of Rostock, Germany; 3 Max-Planck Odense Center on the Biodemography of Aging, University of Southern Denmark, Odense, Denmark; University of California, UNITED STATES

## Abstract

In Biodemography, aging is typically measured and compared based on aging rates. We argue that this approach may be misleading, because it confounds the time aspect with the mere change aspect of aging. To disentangle these aspects, here we utilize a time-standardized framework and, instead of aging rates, suggest the shape of aging as a novel and valuable alternative concept for comparative aging research. The concept of shape captures the direction and degree of change in the force of mortality over age, which—on a demographic level—reflects aging. We 1) provide a list of shape properties that are desirable from a theoretical perspective, 2) suggest several demographically meaningful and non-parametric candidate measures to quantify shape, and 3) evaluate performance of these measures based on the list of properties as well as based on an illustrative analysis of a simple dataset. The shape measures suggested here aim to provide a general means to classify aging patterns independent of any particular mortality model and independent of any species-specific time-scale. Thereby they support systematic comparative aging research across different species or between populations of the same species under different conditions and constitute an extension of the toolbox available to comparative research in Biodemography.

## Introduction

The emerging field of Evolutionary Biodemography seeks answers to pressing questions that arise from challenges brought about by population aging due to the unimpeded and steady increase of human life-expectancy observed over the past century [1]. Striking new insights [2–4] highlight that advances in the field crucially hinge on serviceable, comparative measures of aging across species. To that end, this paper suggests the “shape” of aging as a useful concept and provides measures of the shape of aging that are grounded in demographic theory.

Aging rates are commonly used as a means to compare aging patterns from flies to mice to humans [5, 6], but this approach may be misleading. For a long time comparative biologist have emphasized that, to meaningfully compare survival patterns of species with disparate life spans, time-standardization is required (see [7–11] and in particular [12] for a review). Rates, however, by definition depend on units of time. Such rates could be time-standardized, but yet another challenge arises. To calculate aging rates, gerontologists typically fit parametric functions to mortality data and utilize the function’s parameters to quantify aging. Most commonly the Gompertz function is applied, an exponential mortality model with two parameters: the initial mortality rate, IMR, and the rate of aging, RoA [5]. For human data [13–15] and to some extent also for non-human data [5, 16–18] this is a viable approach, as long as a specific parametric model (e.g. Gompertz-Makeham mortality) provides a good fit to the data. Across a broader range of species, however, goodness of fit and choice of the best model may well differ among populations. Using the same model for all populations could lead to erroneous fit for some of them, while using separate models does not allow meaningful comparison of parameters.

A general, nonparametric approach that accounts for time-standardization is needed to classify the variety of aging patterns. Baudisch [19] suggests such an approach and proposes that, to classify aging, two separate aspects can be distinguished: the pace of life and the shape of aging. Pace captures the timing of death as reflected in the average level of mortality which determines the length of life. In contrast, shape is independent of time and captures the time-(i.e., pace-)standardized change in mortality, i.e., it investigates whether mortality increases or decreases over age and whether it does so mildly or steeply. Thereby, the shape of aging reveals whether organisms get better at escaping death, sustain a certain state, or get worse over their life course, and whether these changes are more or less pronounced. Pace and shape can be considered separate aspects of aging, because it is conceivable that mortality rises or falls over the life course to a lesser or larger extend, independently of whether mortality, on average, is high or low. For example, within a life span of weeks just as well as within a life span of years, mortality may rise (or fall) by a smaller or larger multiple, or mortality may simply stay constant over age, albeit at different levels. What combinations of pace and shape exist depends on the constraints of nature.

Note that other names could be chosen to denote the concept: in analogy to the statistical notion of scale parameters, one could denote the ‘pace of aging’ as the ‘scale of aging’, and the ‘shape of aging’ could be called ‘intensity of aging’ or something similar to emphasize that it quantifies the strength of increase/decrease in mortality. However, since the names ‘pace’ and ‘shape’ have been used in the original paper [19] and several other publications [12, 20], we will hold on to them in this article for the sake of consistency.

The pace-shape approach assigns two values to mortality curves that allow to classify and compare aging patterns, one for pace and one for shape. In a recent study, we have provided a mathematical treatment of pace measures [12]. In this study, we provide a mathematical treatment on how to measure the shape of aging, i.e., how to quantify whether and how much the age-specific hazard of death changes over the course of life. Clearly distinguishing between populations in which, on average, mortality increases (aging), remains constant (sustenance), or decreases (negative aging) over age, shape measures provide important information to investigate conditions that promote vs. inhibit the evolution of aging [20–22].

A notation alert: Demographic quantities are given in continuous formulation here and are defined as follows. The survival function, which gives the probability that death will occur at an age greater than *x*, is denoted as *l*(*x*). It is non-increasing and right-continuous. The force of mortality or age-specific hazard is denoted as *μ* with

$$\begin{array}{c} \mu \left(x\right)=-\frac{l^{\prime }\left(x\right)}{l(x)}. \end{array}$$

Cumulative hazard *H*(*x*) at age *x* is defined as

$$\begin{array}{c} H\left(x\right)=\int_{0}^{x}\mu \left(t\right)dt=-\log\left(l\left(x\right)\right). \end{array}$$

Given a hazard function *μ*, the corresponding survival function is denoted as *l*_*μ*_, i.e.,

$$\begin{array}{c} l_{\mu }\left(x\right)=e^{-\int_{0}^{x}\mu \left(t\right)dt}. \end{array}$$

The probability density function of death is

$$\begin{array}{c} f\left(x\right)=-l^{\prime }\left(x\right)=\mu \left(x\right)l\left(x\right). \end{array}$$ (1)

Remaining life expectancy at age *x* is given by

$$\begin{array}{c} e\left(x\right)=\frac{1}{l(x)}\int_{x}^{\infty }l\left(a\right)da. \end{array}$$

Life expectancy at 0 is denoted by *e*_0_:

$$\begin{array}{c} e_{0}≔e\left(0\right). \end{array}$$

### Pace-standardization

Shape analysis requires measures to be expressed independently of units of time. To eliminate the time dimension, demographic quantities need to be pace-standardized. The approach of pace-standardization is derived, explained and illustrated extensively in [12]; here we restate the most important relationships.

For the analysis in this paper, we will use mean life span (or life expectancy)

$$\begin{array}{c} e_{0}=\int_{0}^{\infty }xf\left(x\right)dx=\int_{0}^{\infty }l\left(x\right)dx, \end{array}$$

as a measure for pace, which, among several potential candidates, has been identified as the most preferable measure [12]. In our study, we only considered cases for which *e*_0_ is finite.

It should be noted that age zero does not have to denote birth; it is simply the age *α* that is assumed to denote the onset of aging (rescaled to a value of 0 via *x̃* = *x*−*α*). The particular choice of *α* depends on the biological circumstances and on what data is available. For example, a reasonable choice of *α* might be the age of reproductive maturity or the age of onset of reproduction.

Chronological age *x* is standardized via

$$\begin{array}{c} x^{\text{s}}=\frac{x}{e_{0}}. \end{array}$$ (2)

Standardized survival curves—functions of standardized age—can then be defined via

$$\begin{array}{c} l^{\text{s}}\left(x^{\text{s}}\right)≔l\left(x\right)=l\left(e_{0}\;x^{\text{s}}\right). \end{array}$$ (3)

Expressions (2) and (3) can be used to derive an expression for the standardized hazard

$$\begin{array}{c} \mu^{\text{s}}\left(x^{\text{s}}\right)=-\frac{1}{l^{\text{s}}\left(x^{\text{s}}\right)}\frac{dl^{\text{s}}\left(x^{\text{s}}\right)}{dx^{\text{s}}}=-e_{0}\frac{1}{l(x)}\frac{dl(x)}{dx}=e_{0}\mu \left(x\right)=e_{0}\mu \left(e_{0}\;x^{\text{s}}\right). \end{array}$$ (4)

The crucial property here is that all standardized survival curves have the same pace value (namely 1):

$$\begin{array}{c} e_{0}^{\text{s}}=\int_{0}^{\infty }l^{\text{s}}\left(x^{\text{s}}\right)dx^{\text{s}}=\frac{\int_{0}^{\infty }l\left(x\right)dx}{e_{0}}=1. \end{array}$$ (5)

Notice that, for the weighted harmonic mean $\bar{\mu }$ of the hazard *μ* with the weights given by the probability density function *f*, it holds that

$$\begin{array}{c} \bar{\mu }=\frac{\int_{0}^{\infty }f\left(x\right)dx}{\int_{0}^{\infty }\frac{1}{\mu (x)}f\left(x\right)dx}=\frac{1}{\int_{0}^{\infty }l\left(x\right)dx}=\frac{1}{e_{0}}. \end{array}$$ (6)

It follows that the standardized hazard results from dividing the unstandardized hazard by the average hazard:

$$\begin{array}{c} \mu^{\text{s}}\left(x^{\text{s}}\right)=\frac{\mu (x)}{\bar{\mu }}. \end{array}$$

Since each demographic function can be expressed in terms of survival *l*, standardized in (3), one can derive standardized expressions for these functions as well:

$$\begin{array}{c} H^{\text{s}}\left(x^{\text{s}}\right)=H\left(x\right),\;f^{\text{s}}\left(x^{\text{s}}\right)=e_{0}f\left(x\right),\;e^{\text{s}}\left(x^{\text{s}}\right)=\frac{e(x)}{e_{0}}. \end{array}$$ (7)

## Shape measures

### Properties of shape measures

In analogy to aging rates, which assign a number to a distribution (e.g. Gompertz) to quantify aging, here we seek a general measure of shape *S*, i.e., a functional *S*:*l* ↦ *S*(*l*) that assigns a real number *S*(*l*) to every survival function *l*. It should capture the quality and degree of change in the force of mortality that takes place over the course of life as measured on the time scale given by pace. From this informal definition, a range of properties follow that any measure of shape *S* should satisfy:

**P1:** *S* should be dimensionless. In particular, it must not depend on time, as opposed to pace measures. This property reflects the fact that shape should be a *relative* quantity which measures how mortality later in life compares to mortality early in life.

**P2:** Two hazard functions that give the same pace-standardized hazard functions also need to give the same value of shape:

$$\begin{array}{c} \mu_{1}^{\text{s}}\left(x^{\text{s}}\right)=\mu_{2}^{\text{s}}\left(x^{\text{s}}\right)\;\;\text{for}\;\text{all}\;x^{\text{s}}\;\Rightarrow \;S\left(l_{\mu_{1}}\right)=S\left(l_{\mu_{2}}\right). \end{array}$$ (8)

This property follows directly from the fact that standardization removes all differences that are due to pace, since all standardized mortality curves have the same pace value. Therefore any difference in shape reflects differences between standardized patterns.

**P3:** The threshold between aging and negative aging for *S* should be zero: If the force of mortality is monotonically increasing, the value of shape should be positive; if the force of mortality is monotonically decreasing, the value of shape should be negative; if the force of mortality is constant, the value of shape should be zero. For differentiable hazard functions, this property is summarized by

$$\begin{array}{c} \frac{d\mu }{dx}\left(x\right)\geq 0\;\;\text{for}\;\text{all}\;x\;\Rightarrow \;S(l_{\mu })\geq 0 \end{array}$$ (9)

$$\begin{array}{c} \frac{d\mu }{dx}\left(x\right)\leq 0\;\;\text{for}\;\text{all}\;x\;\Rightarrow \;S(l_{\mu })\leq 0 \end{array}$$ (10)

$$\begin{array}{c} \frac{d\mu }{dx}\left(x\right)=0\;\;\text{for}\;\text{all}\;x\;\Rightarrow \;S(l_{\mu })=0. \end{array}$$ (11)

Relations (9) and (10) should also hold if the inequalities involved are strict.

This property postulates that zero, being the shape value associated with constant mortality, separates aging (an increase in mortality over age) from negative aging (a decrease in mortality over age) as defined in [21] and [19].

Note that the relevance of this property lies in postulating a clear boundary of aging. This boundary need not be at zero, though given its interpretation of separating postive from negative aging, a value of zero seems most intuitive. In any case, the particular choice of the boundary value only influences the scale of shape values, not the general outcome in ranking how species age.

**P4:** Continuity of *S* with respect to convergence of (standardized) distributions: Let *l* be a survival function and *l*_*n*_ be a sequence of survival functions, so that

$$\begin{array}{c} \lim_{n\to \infty }l_{n}^{\text{s}}\left(x^{\text{s}}\right)=l^{\text{s}}\left(x^{\text{s}}\right)\;\forall x^{\text{s}}\in \left\{t^{\text{s}}:l^{\text{s}}\;\text{is}\;\text{continuous}\;\text{at}\;t^{\text{s}}\right\}. \end{array}$$

Then it should hold that

$$\begin{array}{c} \lim_{n\to \infty }S\left(l_{n}\right)=S\left(l\right). \end{array}$$ (12)

Note that since weak convergence is metrizable via the Lévy metric

$$\begin{array}{c} L\left(l_{1},l_{2}\right)=\inf\left\{s>0:l_{1}\left(x-s\right)-s\leq l_{2}\left(x\right)\leq l_{1}\left(x+s\right)+s\;\forall x\right\}, \end{array}$$

the condition can also be stated as

$$\begin{array}{c} \forall \epsilon >0\;\exists \delta >0:\;L\left(l_{1}^{\text{s}},l_{2}^{\text{s}}\right)<\delta \Rightarrow \left|S\left(l_{1}\right)-S\left(l_{2}\right)\right|<\epsilon . \end{array}$$

**P5:** *S* should satisfy

$$\begin{array}{c} S\leq 1 \end{array}$$

and

$$\begin{array}{c} S\left(l\right)=1\;\Leftrightarrow \;l^{\text{s}}\left(x^{\text{s}}\right)=\left\{\begin{array}{cc} 1 & 0\leq x^{\text{s}}<1,\\ 0 & x^{\text{s}}\geq 1. \end{array}\right. \end{array}$$ (13)

This boundary condition means that the highest possible value of shape (i.e., the value of strongest aging) equals one. The only standardized distribution that this value is assigned to is the one where everybody dies at the same age (i.e., at age *e*_0_ or—in standardized view—at standardized age one). This appeals to intuition since this distribution corresponds to a transition from immortality (nobody dies before a fixed age) to certain death. Naturally, this idealized ‘extreme’ (and non-continuous) distribution will never be observed in real populations, but distributions that approach it can be found in populations of plants or animals, e.g., in semelparous species like Pacific salmon [5], that experience catastrophic death with the onset of reproduction.

Analogous to the choice of zero as the value of shape that marks the boundary of aging, the choice of one as the maximum shape value is arbitrary. There is no strict criterion for doing so. What is important here is to have a value that demarcates the extreme case of maximum aging. The measures above could be scaled so that they point towards infinity for maximum aging as an alternative choice. We prefer a finite value to enable statements about closeness to maximum shape.

One could ask if there is a corresponding lowest value of shape measures. Intuitively, this would have to correspond to a distribution that captures a transition from *μ* = ∞ (certain death) to *μ* = 0 (immortality). Such a transition seems senseless, since if everybody dies at the beginning, there is no one left to be immortal (or, more generally, to experience any sort of aging / change over life). Therefore, we currently do not see a way to meaningfully formalize the notion of a lowest shape value, i.e., of strongest negative aging.

Note that though shape values are meant to quantify in what direction and to what extent mortality changes over life (and thus express a characteristic of *μ*), we choose the survival function *l* to be the basic unit to which a shape value is assigned. We do this mainly because it is possible to construct cases where populations may differ greatly with respect to the level of mortality at high ages, yet little or no differences among such populations could be detected in reality as only a dwindling fraction of individuals would survive to these ages at which differences become apparent. Shape should characterize the aging experience of the “typical”, i.e. average adult individual in the population, because stages that virtually no individual in the population will ever reach can hardly be considered of biologically/evolutionary relevance. A practical reason for considering survival curves is that survival curves are often the primary demographic information available.

This list captures what we consider the most important properties of shape, but it is by no means exhaustive. In fact, we can explicitly name one more condition that is very much in line with the concept of shape:

$$\begin{array}{c} \frac{d\mu_{1}^{\text{s}}}{dx^{\text{s}}}\left(x^{\text{s}}\right)>\frac{d\mu_{2}^{\text{s}}}{dx^{\text{s}}}\left(x^{\text{s}}\right)\;\text{for}\;\text{all}\;x^{\text{s}}\;\Rightarrow \;S\left(l_{\mu_{1}}\right)>S\left(l_{\mu_{2}}\right). \end{array}$$ (14)

This states that if one standardized hazard *μ*_1_ increases more steeply than a second one *μ*_2_ at all ages, then a bigger value of shape is assigned to *μ*_1_ than to *μ*_2_. The main reason this property is not included in the list is simply that for most of the shape measures to be suggested we were not able to prove or disprove that they satisfy it. Therefore, a more elaborate analysis which includes this property (as well as possible further properties) will have to be postponed to future research.

Also, properties that are non-mathematical in nature are not listed here, but can be worthy of consideration. Specifically, ease of use comes to mind, as further elaborated in the ‘What measure(s) to use?’-section later on.

### “Candidates” for shape measures

Below, seven different demographic quantities are listed that satisfy all or some of the properties in the previous section and thus are potential candidate measures that can capture the shape of aging. We chose only measures that rely on established and demographically meaningful quantities.

**Measure 1**: Mortality ratio

$$\begin{array}{c} S_{1}\left(l\right)=1-\frac{\mu (0)}{\mu (e_{0})}. \end{array}$$ (15)

Baudisch [19] suggested the ratio $\frac{\mu (e_{0})}{\mu (0)}$ as a potential shape measure, and *S*_1_ simply rescales this ratio in order to ensure that the boundary value is 1 and the threshold between aging and negative aging is zero. This ratio is based on the simple idea of comparing the force of mortality at a later age to the force of mortality at an earlier age in order to quantify whether and how much the hazard increases/decreases over age. The disadvantage of this measure is its dependence on the value of *μ* at two particular ages and the resulting lack of robustness. Also, since the value of *μ* at all other ages is not taken into account (except for the effect on *e*_0_), the information content of this measure is rather low. Therefore, it is useful mainly for smooth and monotonic hazard functions. Note that a similar measure (mortality rate at age 80 over the mortality rate at age 30) was used in [23] for human data.

**Measure 2**: Average of $\frac{d\mu }{dx}$ with respect to *l*(*x*)

$$\begin{array}{c} S_{2}\left(l\right)=1-e^{-D} \end{array}$$ (16)

with

$$\begin{array}{c} D=e_{0}\int_{0}^{\infty }\frac{d\mu }{dx}\left(x\right)l\left(x\right)dx=\int_{0}^{\infty }\frac{\mu (x)-\mu (0)}{\bar{\mu }}f\left(x\right)dx. \end{array}$$

This measure is based on the idea that in order to get a global value *D* which indicates direction and strength of the change in *μ*, one can sum up the local values of the derivative $\frac{d\mu }{dx}$, weighted by the proportion of the population still alive at each age (and multiply the result by *e*_0_ to pace-standardize). As follows from a relationship derived by Vaupel [24], this is equal to the average lifetime change in *μ* (in this case divided by average mortality). In (16), *D* is then rescaled to ensure sure that the boundary value is 1.

As an improvement to measure *S*_1_, this measure takes into account the whole hazard function. However, it is still not robust to changes in *μ*(0).

**Measure 3**: Probability to survive up to the mean age at death *e*_0_

$$\begin{array}{c} S_{3}\left(l\right)=1-H\left(e_{0}\right)=1+\log\left(l\left(e_{0}\right)\right). \end{array}$$ (17)

This simple measure uses the log-transform of *l*(*e*_0_), the probability to survive up to the mean length of life (or in our terminology: to the value of pace), and is based on the observation that cumulative hazard *H*(*e*_0_) = −log(*l*(*e*_0_)) at *e*_0_ is smaller than one for increasing hazard functions and bigger than one for decreasing hazard functions. A heuristic ‘proof’ of this fact is that

$$\begin{array}{c} H\left(e_{0}\right)=H^{\text{s}}\left(1\right)=\int_{0}^{1}\mu^{\text{s}}\left(x^{\text{s}}\right)dx^{\text{s}}, \end{array}$$

which gives an average value of *μ* ^s^ between 0 and 1. Compared to the overall average $\bar{\mu^{\text{s}}}=1$ (see (6) and (5)), this should reflect whether *μ* increases or decreases over age. However, this is not a real proof since the averaging is done differently for $\bar{\mu^{\text{s}}}$ according to (6). A strict proof can be found in S1 Appendix. The measure still has the disadvantage of lacking a good interpretation in terms of aging. Nevertheless, *S*_3_ might be preferred in some situations, since it is easy to calculate. The unscaled form of this measure, *l*(*e*_0_), was used in a recent study on the shape of aging in angiosperms [20].

In order to find other measures of shape, one might also look at the schedule of remaining life-expectancy at age *x*, *e*(*x*), rather than *μ*(*x*), since their respective behaviours mirror each other in the following sense. Consider a generalization of (6):

$$\begin{array}{c} \frac{1}{e(x)}=\frac{\int_{x}^{\infty }w\left(t\right)dt}{\int_{x}^{\infty }\frac{1}{\mu (t)}w\left(t\right)dt}≕\bar{\mu }\left(x\right)\;\text{with}\;w\left(t\right)=\frac{f(t)}{l(x)}, \end{array}$$ (18)

so that $\bar{\mu }(x)$ is the weighted harmonic mean of *μ* for ages ≥ *x* with weighting function *w* (note that $\int_{x}^{\infty }w(t)dt=1$). The pattern of the age-specific average hazard $\bar{\mu }(x)=1/e(x)$ is a convenient continuous replacement for *μ*(*x*). In particular, a monotonically increasing *μ*(*x*) implies a monotonically decreasing *e*(*x*), and a monotonically decreasing *μ* implies a monotonically increasing *e*(*x*). This reasoning motivates the following measures, which are based on *e*(*x*).

**Measure 4**: Life expectancy ratio

$$\begin{array}{c} S_{4}\left(l\right)=1-\frac{e(e_{0})}{e_{0}}. \end{array}$$ (19)

This measure is based on the same idea as *S*_1_, but with *e*(*x*) instead of *μ*(*x*): Take the value of *e* at a later age and compare it to the value of *e* at age 0. In principle, any later age could be compared to any earlier age. However, aiming for a consistent and meaningful combination of shape and pace measures suggests that choosing the value of pace—the characteristic life span—as ‘later age’ is most reasonable.

**Measure 5**: Life table entropy

$$\begin{array}{c} S_{5}\left(l\right)=1-\frac{e^{†}}{e_{0}}=1-\bar{H}. \end{array}$$ (20)

Here,

$$\begin{array}{c} e^{†}=\int_{0}^{\infty }e\left(x\right)f\left(x\right)dx=\int_{0}^{\infty }H\left(x\right)l\left(x\right)dx=\int_{0}^{\infty }-\log\left(l\left(x\right)\right)l\left(x\right)dx \end{array}$$

denotes remaining life expectancy lost due to death and the ratio

$$\begin{array}{c} \bar{H}=\frac{e^{†}}{e_{0}} \end{array}$$

is called entropy—note that in order to be able to calculate $\int_{0}^{\infty }H(x)l(x)dx$, the value of *H*(*x*)*l*(*x*) for *l*(*x*) = 0 is defined to equal lim_*x*↘0_ *x*log(*x*), which according to the rule of l’Hôpital is 0. Thus, *S*_5_ measures the average change in expected life span between age *zero* and death. The idea behind using (20) as a shape measure is that for an increasing hazard function (and thus decreasing schedule of *e*(*x*)), average remaining life expectancy at death must be lower than *e*_0_ (and vice versa for decreasing hazard). This is reflected even more clearly when applying integration by parts to (20) (see [24]):

$$\begin{array}{c} S_{5}\left(l\right)=-\frac{1}{e_{0}}\int_{0}^{\infty }\frac{de}{dx}\left(x\right)l\left(x\right)dx. \end{array}$$ (21)

This means that in a stationary population, *S*_5_ measures the total incremental change in *e*(*x*) with age.

*e* ^†^ and entropy $\bar{H}$ are quantities frequently used in demography for different purposes. *e* ^†^, introduced in [25], is usually used as a measure of life disparity [26]. Life table entropy $\bar{H}$ is the elasticity of life expectancy with respect to a proportional change in mortality [27, 28] and has been used in population biology [29–31]. The notation $\bar{H}$ was suggested by J.W. Vaupel (personal communication) because $\bar{H}$ can be viewed as the average value of the cumulative hazard function *H*(*x*). It has been noted [30] that $\bar{H}$ can be used for the classification of survival curves in the sense of Pearl and Miner [8], which is related to the concept of shape, so that *S*_5_ qualifies as a potential shape measure.

**Measure 6**: Coefficient of variation

$$\begin{array}{c} S_{6}\left(l\right)=1-\frac{\sigma }{e_{0}}=1-c_{v} \end{array}$$ (22)

with

$$\begin{array}{c} \sigma =\sqrt{\int_{0}^{\infty }{\left(x-e_{0}\right)}^{2}f\left(x\right)dx} \end{array}$$

being the standard deviation of the age at death and

$$\begin{array}{c} c_{v}=\frac{\sigma }{e_{0}} \end{array}$$

denoting the corresponding coefficient of variation. Variance *σ* ^2^ is often used as a measure of life span inequality [32–34].

This measure is motivated by the fact that the variance in the age at death equals the average squared remaining life expectancy at death [35]:

$$\begin{array}{c} \sigma^{2}=\int_{0}^{\infty }e^{2}\left(x\right)f\left(x\right)dx. \end{array}$$

Thus, the same logic as in the case of *S*_5_ applies: for an increasing hazard function (and thus decreasing *e*(*x*)), squared life expectancy at death must be lower than $e_{0}^{2}$ (and vice versa for decreasing hazard). In this sense, it is a variation of measure *S*_5_ with a different way of finding the average.

**Measure 7**: A variant of the Gini coefficient

$$\begin{array}{c} S_{7}\left(l\right)=2\frac{\int_{0}^{\infty }l^{2}\left(x\right)dx}{\int_{0}^{\infty }l\left(x\right)dx}-1=\frac{2}{e_{0}}\int_{0}^{\infty }l^{2}\left(x\right)dx-1. \end{array}$$ (23)

Hanada [36] has shown that

$$\begin{array}{c} \frac{\int_{0}^{\infty }l^{2}\left(x\right)dx}{\int_{0}^{\infty }l\left(x\right)dx}=1-G, \end{array}$$ (24)

where *G* denotes the Gini coefficient, so that *S*_7_(*μ*) = 1−2*G*. *G* is originally defined as a property of the Lorenz curve, and it is commonly used for measuring and analysing inter-individual inequality in the length of life. It can be shown that it equals the mean of the absolute differences in individual ages at death relative to the average length of life [37, 38] and takes values between 0 (perfect equality) and 1 (perfect inequality). Note that (23) can also be expressed in a different way: using integration by parts and the fact that *de*(*x*)/*dx* = *e*(*x*)*μ*(*x*)−1, it can be shown (see S1 Appendix) that

$$\begin{array}{c} S_{7}\left(l\right)=1-\frac{2}{e_{0}}\int_{0}^{\infty }e\left(x\right)l\left(x\right)f\left(x\right)dx=-\frac{1}{e_{0}}\int_{0}^{\infty }\frac{de}{dx}\left(x\right)l^{2}\left(x\right)dx. \end{array}$$ (25)

The right hand side is similar to (21), only with a different weight function, and expresses more clearly why *S*_7_ is suited to quantify aging. Also, consider that

$$\begin{array}{c} \int_{0}^{\infty }l^{2}\left(x\right)dx=\int_{0}^{\infty }e^{-\int_{0}^{x}2\mu \left(t\right)dt}dx \end{array}$$ (26)

is the life expectancy that results from a doubling of the hazard *μ* at all ages, so that *S*_7_ relates to perturbation theory. Thus, in this respect it is also similar to *S*_5_.

## Discussion

### What measure(s) to use?

What measures of shape are suitable and preferable for comparative research on mortality patterns? From a theoretical perspective, **P1**–**P5** provide the basis to identify (a) preferable candidate(s). Table 1 summarizes whether properties **P1**–**P5** are satisfied by measures *S*_1_–*S*_7_ (for mathematical proofs see S1 Appendix). Only measure *S*_7_ satisfies all properties, while measures *S*_4_–*S*_6_ satisfy all but one. As far as a hierarchy of the properties is concerned, the most important properties are **P1**–**P3** and **P5**, because they formalize the main principles of the concept of shape: shape measures are time-independent estimators of the degree of change in pace-standardized mortality over age. Therefore it can be concluded that, to quantify shape, measures *S*_1_–*S*_3_ are suited reasonably well (conditional on the caveats mentioned after their introduction) as they satisfy the first three properties, and measures *S*_4_–*S*_7_ are suited best. To further distinguish among the latter measures, consider that *S*_4_–*S*_6_ are calculated based on the conditional expectation of the amount of time lived after age *x* (i.e., life expectancy *e*(*x*)), while *S*_7_ rests on the *unconditional* expectation of the amount of time lived after age *x* (i.e., *e*(*x*)*l*(*x*); see equation (25) and [39]). This helps understand why *S*_4_–*S*_6_ do not satisfy **P4**, while *S*_7_ does. A few outliers with very long life spans will have a bigger impact on *S*_4_–*S*_6_ than on *S*_7_. Since we view shape as a population concept, i.e., expressing the experience of an average individual in the population, **P4** is an appropriate property of continuity, and from this perspective, *S*_7_ is the best shape measure. However, for some applications it may be desirable to use a measure that is more sensitive to individual diversity in life spans. In such cases, measures *S*_4_–*S*_6_ would be a better choice.

**Table 1:** Properties and measures.

	**P1**	**P2**	**P3**	**P4**	**P5**
*S*_1_	yes	yes	yes	no	no
*S*_2_	yes	yes	yes	no	no
*S*_3_	yes	yes	yes	no	no
*S*_4_	yes	yes	yes	no	yes
*S*_5_	yes	yes	yes	no	yes
*S*_6_	yes	yes	yes	no	yes
*S*_7_	yes	yes	yes	yes	yes

Summary of whether shape measures *S*_1_–*S*_7_ satisfy properties **P1**–**P5**.

**Fig 1:**
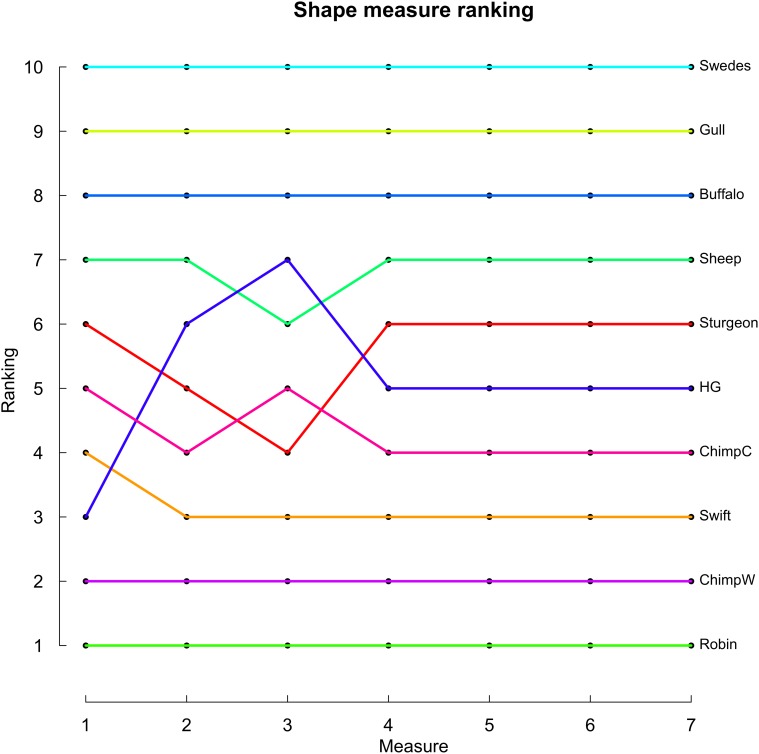
Comparison of rankings as provided by the different shape measures. For the ten populations used in [19] (HG stands for hunter-gatherers, ChimpW for wild chimpanzees, ChimpC for captive chimpanzees), the ranking of shape values (from lowest to highest value) is similar for all of the shape measures *S*_1_–*S*_7_.

From an empirical perspective the question arises whether different measures lead to different outcomes when classifying aging patterns as more or less senescent. Baudisch [19] investigated the matter using preliminary measures by extracting mortality parameters for different species (from maturity onwards), calculating pace and shape values and depicting them in pace-shape space. For consistency with this analysis, we use the same data and calculate shape values based on the measures derived above, including 10 populations that comprise birds, fish, mammals, captive and wild chimps, as well as hunter gatherers and Swedish females of 2007 (see the original paper [19] for more details). With this analysis, we do not aim to provide an empirical pace shape study (see e.g. [20]), but to illustrate how to utilize the shape values derived in this paper and to investigate whether the choice of a measure makes a difference. The data stems from smooth and simple parametric models, and thus sample size should not be the source of any drastic distortions in the results; future research will have to take a more detailed look at how our shape measures are affected by sample size, especially if they are calculated without a previous smoothing of the data. Fig. 1 serves to get an impression of the empirical perfomance of alternative measures. It shows how species rank with respect to the different shape measures (higher rank ≅ higher shape value). Additionally, Table 2 gives the Spearman’s ranking coefficients. Both Fig. 1 and Table 2 reveal that alternative shape measures *S*_1_–*S*_7_ are highly correlated. Therefore, at least judging from this small data set, the choice of a particular shape measure seems not crucial (in particular, measures *S*_4_, *S*_5_, *S*_6_ and *S*_7_ are perfectly correlated). In the light of this result it could also be concluded that the most simple and easy to use measures, which are *S*_1_ and *S*_3_, should be preferred, because the extra work necessary to calculate the other measures is not reflected in a significantly different outcome of analysis (again with the caveat that we have only used a small dataset with rather well-behaved age-patterns of mortality here).

**Table 2:** Spearman’s rank correlation coefficients.

	*S*_1_	*S*_2_	*S*_3_	*S*_4_	*S*_5_	*S*_6_	*S*_7_
*S*_1_	1.00						
*S*_2_	0.93	1.00					
*S*_3_	0.87	0.98	1.00				
*S*_4_	0.96	0.99	0.94	1.00			
*S*_5_	0.96	0.99	0.94	1.00	1.00		
*S*_6_	0.96	0.99	0.94	1.00	1.00	1.00	
*S*_7_	0.96	0.99	0.94	1.00	1.00	1.00	1.00

Spearman’s rank correlation coefficients of the values of shape measures *S*_1_–*S*_7_ applied to the data of ten populations taken from [19].

Thus accounting for both theoretical and empirical arguments, we conclude that *S*_4_–*S*_7_ provide servicable and useful shape measures. Note that Baudisch [19] also suggested two measures based on longevity, denoted as Ω, the age when 99% of the adult population has died, which is an approximation to the longest life span observed. Such measures are worth investigating, but due to statistical difficulties in obtaining Ω, measures based on Ω are less preferable, especially when sample sizes are low, and thus will not be discussed here.

### The advantage of using shape measures

Coming back to the concern that aging rates may be misleading when comparing aging patterns among populations that differ in life span, here we illustrate this problem by means of a simple theoretical example.

**Fig 2:**
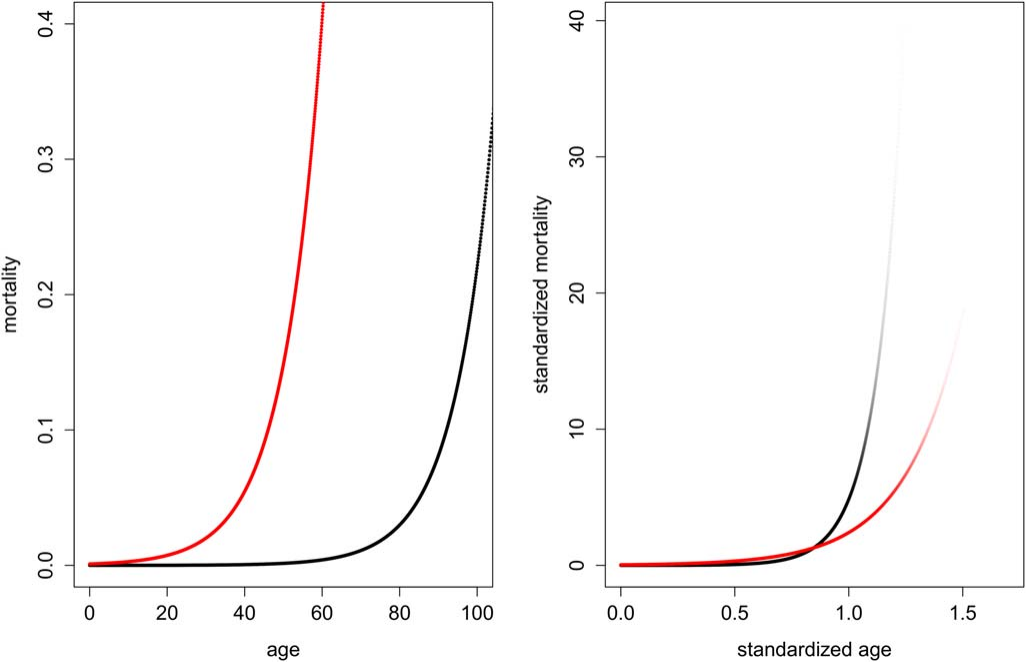
The effect of pace standardization on age-specific hazard can reverse conclusions. Gompertz mortality *μ*(*x*) = *ae* ^0.1*x*^ with *a* = 0.00001 (black line) and *a* = 0.001 (red line). Left: unstandardized perspective. Right: standardized perspective. The intensity of the colors is proportional to the value of the respective survival function. If the strength of aging is measured by how steeply age-specific hazard increases over life, the unstandardized perspective implies that the population with *a* = 10^−3^ experiences stronger aging than the population with *a* = 10^−5^; however, if the difference in life span is accounted for, i.e., if the hazard curves are standardized, the conclusion is reversed.

Assume a Gompertz force of mortality

$$\begin{array}{c} \mu \left(x\right)=ae^{bx} \end{array}$$

with a fixed parameter *b* = 0.1 and two different values of parameter *a*, say *a*_1_ = 0.00001 and *a*_2_ = 0.001. The age-specific hazard curves are plotted in Fig. 2. The black curves denote *μ*_1_, the red curves denote *μ*_2_. The picture on the left shows the usual unstandardized perspective, while the picture on the right shows standardized mortality over standardized age (see equations (2) and (4)). Additionally, in the picture on the right the intensity of the colors is proportional to the value of the respective survival function, i.e. the curves fade out as the number of survivors approaches zero.

The unstandardized perspective suggests that the increase in *μ*_2_ over age is steeper than the increase in *μ*_1_. Commonly, this observation would be interpreted to show that a population subject to *μ*_2_ experiences stronger aging than a population subject to *μ*_1_. Alternatively, one may interpret the fact that both populations have the same aging rate of *b* = 0.1 to mean that both populations age the same. However, a pace-shape perspective reveals a different scenario. If the force of mortality is depicted over the time scale provided by average life span, i.e. pace, the conclusion changes: the standardized perspective shows that *μ*_2_ is increasing less over the course of (average) life than *μ*_1_. Thus, if the difference in life span is accounted for, which implies that the number of survivors suffering a given level of *μ* at any age is taken into account, a population subject to *μ*_2_ actually experiences *weaker* aging than a population subject to *μ*_1_.

This result, based on visual inspection of the standardized mortality curves, is corroborated by Table 3. The shape values reveal that, indeed, population two is always classified by lower values than population one. Though population one experiences a slower pace of life than population two, it experiences a steeper shape of aging. Therefore we argue that the shape measures introduced in this paper provide a more accurate assessment of the strength of aging over the course of life than the value of the ‘rate of aging’ *b*.

**Table 3:** Shape values for *μ*_1_ and *μ*_2_.

	**S1**	**S2**	**S3**	**S4**	**S5**	**S6**	**S7**
*μ*_1_	0.999	0.999	0.438	0.900	0.884	0.851	0.839
*μ*_2_	0.983	0.983	0.419	0.795	0.764	0.710	0.678

Shape values assigned to *μ*_1_ and *μ*_2_ by the measures *S*_1_–*S*_7_. Note that the shape values assigned to *μ*_2_ are consistently smaller than the shape values assigned to *μ*_1_, which classify population two as showing weaker aging than population one.

More importantly, shape measures do not depend on a particular parametric mortality model. Providing a non-parametric approach that clearly distingushes the pace of life from the pattern of aging is a major advantage of the framework presented here.

### Scaling of shape measures

The shape values derived in this paper are meaningful for ranking populations with respect to how much they age. Direct interpretation of their specific numerical values, i.e., the (relative or absolute) distance in shape values between populations, is not meaningful. This is the price to be paid for scaling all shape measures to share a common threshold value (in our case zero) and a common value of strongest senescence (in our case one). This has been done for easy comparability between the measures.

However, for empirical applications it may be desirable to work with rescaled shape measures to allow for direct interpretation of their values. S2 Appendix provides a list of simple transformations that allow to rescale measures *S*_1_–*S*_7_ to ratios of late-life mortality over early-life mortality, which enable a direct quantification of the shape of age-specific hazard.

## Related results and perspectives

Previous attempts to classify aging patterns on a standardized time scale have distinguished survival curves on a qualitative basis. Based on visual inspection, Pearl and Miner [7, 8] suggested grouping survival curves into three types—rectangular, linear, and L-shaped—that are associated with increasing, constant and decreasing death rates respectively, a notion picked up subsequently by Deevey [9, 10]. The shape approach quantifies this rough classification and embeds it in a framework that also integrates the pace of aging [12]. Different to previous approaches, the pace-shape approach suggests a quantitative framework providing general measures of aging with direct demographic interpretations.

The shape of aging as investigated in this article is methodologically related to a concept already established in demography: mortality compression / rectangularization of the survival curve. The common ground with shape is that researchers on mortality compression are often interested in measuring how ‘distant’ a given distribution is perceived to be from the boundary distribution where everybody dies at the same age (i.e., the survival curve is rectangular and there is no variability of age at death), which in terms of shape corresponds to strongest aging, i.e. going from immortality to certain death (see property **P5**). Researchers have developed several measures to quantify the rectangularity of survival, and it has been recognized that the average length of life is in general independent of its variability, just as pace is independent of shape (in the sense that *in principle* all combinations of pace and shape values are possible; for a given set of populations however, there might be a correlation between pace and shape values, and the researcher’s task is to ask why)—see [40] and [41] for a good overview of the topic. In particular, [40] evaluates ten measures of rectangularity, among them Keyfitz’s $\bar{H}$, standard deviation *σ* and Gini coefficient, and these quantities are the basis for our shape measures *S*_5_–*S*_7_. The approach of (graphically) comparing a longevity measure and a compression measure that has been used in this area of research (see Figures Seven and Nine in [40] and Figures Three and Five in [42]) can be considered to be a predecessor of the pace-shape approach, in particular of the ‘pace-shape space’ (see two paragraphs below).

There are however major differences between rectangularization and the concept of shape: Shape is primarily concerned about strength of aging (i.e., the steepness of increase/decrease in mortality), not about rectangularity, and the rectangular survival pattern just happens to occur in the case of strongest aging. From a formal perspective, rectangularization is generally based on the probability density function *f*(*x*) (strongly influenced by the statistical framework of central tendency and dispersion), whereas shape is based on hazard *μ*(*x*). Also, in rectangularization research there is no standardization with respect to population-specific time scales. Generally speaking, research on rectangularization is focused on describing and explaining trends in human mortality, while the concept of pace and shape is primarily meant for biodemographers who study and compare aging in different species.

**Fig 3:**
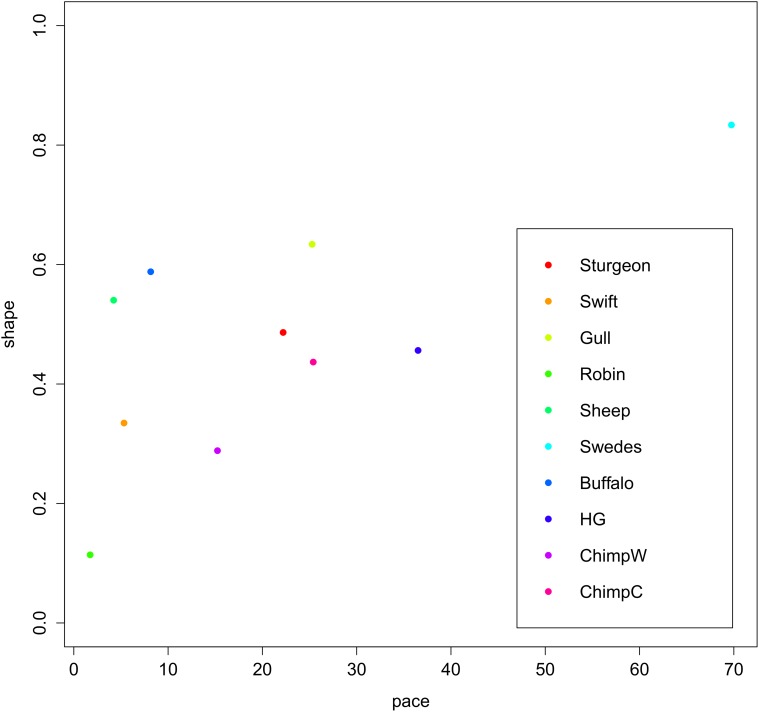
Species in pace-shape space. Pace values (as measured by life expectancy *e*_0_) on the x-axis versus shape values (as measured by *S*_7_) on the y-axis for ten populations taken from [19] (HG stands for hunter-gatherers, ChimpW for wild chimpanzees, ChimpC for captive chimpanzees).

There are several directions for future studies based on the method presented in this paper. The most central application of the pace and shape framework and the measures we derived in this paper is for the systematic comparison of hazard functions between different species or between populations of the same species that experience different life conditions. The method is particularly useful when data for many populations is to be compared, since depicting hazard functions as points in a pace-shape space (pace on the x-axis, shape on the y-axis) allows for a clear overview over the aging-related features of these schedules. This is illustrated in Fig. 3 with the species used in the original paper by Baudisch [19]; pace is measured by *e*_0_, shape is measured by *S*_7_. Even though the shape measure we use is more sophisticated than the simple preliminary measure used by Baudisch, the results are similar to what could be read from the original figure: species can be found in all areas of the diagram except for the combination slow (pace)-weak (shape), and modern humans (Swedes) have both exceptionally big values of both pace and shape.

Furthermore, research on the effects of certain treatments (like dietary restrictions etc. [43–45]) on aging patterns might gain new impulses from the pace-shape framework, since so far it mostly relies on the use of life span (i.e., pace) measures or specific parameters like Gompertz *b* [5, 16, 44, 46]. Whether or not the shape (by the definition used here) is affected—and if yes, how—is worth investigating by means of pace standardization and one or several of the measures *S*_1_–*S*_7_.

The framework developed here focuses on mortality, but it is recognized that aging is also expressed in diminishing reproductive output [4, 47]. A natural extension of the current method would thus be to consider not only age-specific hazard, but also quantities capable of capturing reproductive aging, e.g. Fisher’s reproductive value (see Partridge in [48]) or fertility rates. This is an important direction for future research, and the extension of the framework to the additional dimension of fertility raises many important questions. How to measure pace and shape of reproductive aging? Do we treat mortality and fertility separately, or do we prefer measures that use a combination of both (such as generation time)? How to incorporate menarche and menopause into the analysis? A systematic investigation on this issue is required, and it would preferably follow the method laid down in this paper (suggestion of desired properties, suggestion of potential measures, evaluation).

Another important line of follow-up research is to investigate how shocks/perturbations of the underlying mortality are reflected in the shape of aging. For pace, or at least for the important pace measure *e*_0_, the effect of perturbations has already been studied [49], and it would be highly desirable to carry out a similar investigation for one or several shape measures.

We emphasize that reducing the complexity of a hazard function (or any other age-dependent function) to the two dimensions of pace and shape implies a loss of information. Therefore this method should be viewed as a means of ‘zooming out’ to provide a rough landscape for better orientation; after this orientation has been gained, zooming in to look at the details of the full (standardized) hazard functions can be done depending on the research question. In this sense, the shape measures introduced in this paper are a sound starting point for non-parametric comparative research on the shape of aging, and they provide a valuable extension to the mathematical toolbox available to biodemographers.

## Supporting Information

S1 Appendix Mathematical proofs. (PDF)

S2 Appendix Rescaling of shape measures. (PDF)
