# Supplementary material for: Quantifying the Shape of Aging
Source: PLoS One. 2015 Mar 24;10(3):e0119163. doi: 10.1371/journal.pone.0119163 (PMC4372288; doi:10.1371/journal.pone.0119163)
Supplement: S1 Appendix — (PDF) [file pone.0119163.s001.pdf]

PLOS

S1 Appendix

Here we check whether measures $S_1 - S_6$ satisfy properties **P1-P5** and thus are good candidates for shape measures.

Measure 1:

$$S_1(l) = 1 - \frac{\mu(0)}{\mu(e_0)}.$$

P1: Satisfied by definition.

P2: Satisfied because

$$S_1(l) = 1 - \frac{\mu^{\mathrm{s}}(0)}{\mu^{\mathrm{s}}(1)}.$$

P3: Satisfied since $\mu(0) \leq \mu(e_0)$ if μ rises over age, $\mu(0) = \mu(e_0)$ if μ is constant, and $\mu(0) \geq \mu(e_0)$ if μ falls over age.

P4: Not satisfied since the measure is sensitive to changes of μ at 0 and e_0, while such changes are not necessarily reflected in the survival function.

P5: $S_1 \leq 1$ is satisfied by definition, but the remainder of the property is not satisfied because $\mu(0) = 0$ implies $S_1(l) = 1$, but the corresponding survival curve does not have to be the one of strongest senescence.

Measure 2:

$$S_2(l) = 1 - e^{-D}$$

with

$$D = e_0 \int_0^\infty \frac{d\mu}{dx}(x) l(x) dx = \int_0^\infty \frac{\mu(x) - \mu(0)}{\bar{\mu}} f(x) dx.$$

P1: Satisfied by definition.

P2: Satisfied because

$$D = \int_0^\infty (\mu^{\mathrm{s}}(x^{\mathrm{s}}) - \mu^{\mathrm{s}}(0)) f^{\mathrm{s}}(x^{\mathrm{s}}) dx^{\mathrm{s}}.$$

P3: Satisfied: D is a weighted average of $\frac{d\mu}{dx}$, and $1 - e^{-D}$ is bigger than 0 if D is bigger than 0, 0 if $D = 0$, and smaller than 0 if D is smaller than 0.

P4: Not satisfied since the measure is sensitive to changes in μ at 0, while such changes are not necessarily reflected in the survival function.

P5: $S_2 \leq 1$ is satisfied by definition, but the remainder of the property is not satisfied, because

$$l_U(x^{\mathrm{s}}) = \begin{cases} 1 & x^{\mathrm{s}} < 1, \\ 0 & x^{\mathrm{s}} \geq 1, \end{cases}$$

does not correspond to any real hazard function (since it would require $\mu^{\mathrm{s}}(1) = \infty$).

Measure 3:

$$S_3(l) = 1 - H(e_0) = 1 + \log(l(e_0))$$

P1: Satisfied by definition.

P2: Satisfied because $l(e_0) = l^{\mathrm{s}}(1)$.

P3: Satisfied: The scaling makes sure that the measure takes values lower or equal to 1, and for constant hazard

$$H(e_0) = \int_0^{e_0} \mu(t)dt = \mu e_0 = 1,$$

so that $S_3(\mu) = 0$. Furthermore, if μ is increasing, $H(x)$ is a convex function, so that Jensen's inequality yields

$$H(e_0) \leq \int_0^{\infty} H(x)f(x)dx = \int_0^{\infty} \mu(x)l(x)dx = 1,$$

which means $S_3(\mu) \geq 0$. Similarly, if μ is decreasing, $H(x)$ is a concave function, so that Jensen's inequality yields

$$H(e_0) \geq \int_0^{\infty} H(x)f(x)dx = \int_0^{\infty} \mu(x)l(x)dx = 1,$$

which means $S_3(\mu) \leq 0$.

P4: Not satisfied. Consider Gompertz mortality with $b = 1$: $\mu(x) = ae^x$. Then

$$e_0 = e^a \int_a^{\infty} \frac{e^{-z}}{z} dz = e^a E_1(a)$$

with $E_1(a) = \int_a^{\infty} \frac{e^{-z}}{z} dz$. For small a this can be approximated by

$$e_0 \approx e^a(-\gamma - \log(a))$$

with $\gamma = 0.57721\ldots$ denoting the Euler-Mascheroni constant (see [1]; specifically in demographic context, this is a special case of eq.(9) in [2], eq.(11) in [3] , eq.(9) in [4] and eq.(6) in [5]).

It follows that for small a

$$l^{\mathrm{s}}(x^{\mathrm{s}}) = e^{a\left(1-e^{x^{\mathrm{s}} e_0}\right)} \approx e^{a\left(1-e^{x^{\mathrm{s}} e^a(-\gamma-\log(a))}\right)} \approx e^{a\left(1-x^{\mathrm{s}} e^a\right)} e^{-e^{-x^{\mathrm{s}}\gamma e^a}}$$

Since

$$\lim_{a\to 0} e^{-x^{\mathrm{s}}\gamma e^a} = e^{-x^{\mathrm{s}}\gamma}$$

and

$$\lim_{a\to 0} a^{(1-x^{\mathrm{s}} e^a)} = \begin{cases} 0 & x^{\mathrm{s}} < 1, \\ 1 & x^{\mathrm{s}} = 1, \\ \infty & x^{\mathrm{s}} > 1, \end{cases}$$

it follows that choosing a sequence $(a_n)_{n\in\mathbb{N}}$ with $a_n \to 0$ implies

$$l_n^{\mathrm{s}}(x^{\mathrm{s}}) \to \begin{cases} 1 & x^{\mathrm{s}} < 1, \\ e^{-e^{-\gamma}} & x^{\mathrm{s}} = 1, \\ 0 & x^{\mathrm{s}} > 1. \end{cases}$$

This means that $(l_n^{\mathrm{s}})_n$ weakly converges to the survival function of strongest aging

$$l_U(x^{\mathrm{s}}) = \begin{cases} 1 & x^{\mathrm{s}} < 1, \\ 0 & x^{\mathrm{s}} \geq 1, \end{cases}$$

since convergence is required only for ages at which l_u is continuous, i.e., for $x^{\mathrm{s}} \neq 1$. But

$$S_3(l_n) = 1 + \log(l_n^{\mathrm{s}}(1)) \to 1 - e^{-\gamma} < 1,$$

so the convergence of l_n^{s} to l_U does not imply $S_3(l_n) \to S_6(l_U) = 1$.

P5: By definition, $S_3 \leq 1$, but the remainder of the property is not satisfied since $S_3(l) = 1$ implies $l^{\mathrm{s}}(1) = 1$, so that $l^{\mathrm{s}} \neq l_U$ with

$$l_U(x^{\mathrm{s}}) = \begin{cases} 1 & x^{\mathrm{s}} < 1, \\ 0 & x^{\mathrm{s}} \geq 1. \end{cases}$$

Measure 4: Life expectancy ratio

$$S_4(l) = 1 - \frac{e(e_0)}{e_0}. \tag{1}$$

P1: Satisfied by definition.

P2: Satisfied because $S_4(l) = 1 - e^{\mathrm{s}}(1)$.

P3: Satisfied. If μ is increasing, e is decreasing and thus $e(e_0) \leq e_0$. If μ is decreasing, e is increasing and thus $e(e_0) \geq e_0$. If μ is constant, then so is e and thus $e(e_0) = e_0$.

P4: Not satisfied. For each $1 \leq n \in \mathbb{N}$ there exists a continuous standardized survival curve l_n^{s} (i.e., $\int_0^\infty l_n^{\mathrm{s}}(x^{\mathrm{s}})dx^{\mathrm{s}} = 1$) satisfying the following two properties $\forall n \in \mathbb{N}$:

- $l_n^{\mathrm{s}}(1) = 2^{-2^{2n}}$
- $\int_1^\infty l_n^{\mathrm{s}}(x^{\mathrm{s}})dx^{\mathrm{s}} = 2^{-n}$.

The specific values of l_n^{s}, other then for $x^{\mathrm{s}} = 1$, are not important; it only matters that they can be chosen so that all the l_n^{s} satisfy the properties (which is the case since the properties do not contradict $\int_0^\infty l_n^{\mathrm{s}}(x^{\mathrm{s}})dx^{\mathrm{s}} = 1$). Then it holds that $\lim_{n\to\infty} l_n^{\mathrm{s}}(x^{\mathrm{s}}) = l_U(x^{\mathrm{s}})$ $\forall x^{\mathrm{s}}$ with

$$l_U(x^{\mathrm{s}}) = \begin{cases} 1 & x^{\mathrm{s}} < 1, \\ 0 & x^{\mathrm{s}} \geq 1. \end{cases}$$

For $x^{\mathrm{s}} \geq 1$, this follows from $l_n^{\mathrm{s}}(x^{\mathrm{s}}) \leq l_n^{\mathrm{s}}(1)$ and $l_n^{\mathrm{s}}(1) = 2^{-2^{2n}}$, which converges to 0 for $n \to \infty$. For any fixed $x^{\mathrm{s}} < 1$, assume there existed a $\epsilon > 0$ so that $\forall N \in \mathbb{N}\ \exists n \geq N$ with $l_n^{\mathrm{s}}(x^{\mathrm{s}}) \leq 1 - \epsilon$. Then

$$\int_0^1 l_n^{\mathrm{s}}(t^{\mathrm{s}})dt^{\mathrm{s}} = \int_0^{x^{\mathrm{s}}} l_n^{\mathrm{s}}(t^{\mathrm{s}})dt^{\mathrm{s}} + \int_{x^{\mathrm{s}}}^1 l_n^{\mathrm{s}}(t^{\mathrm{s}})dt^{\mathrm{s}} \leq$$
$$\leq x^{\mathrm{s}} + (1-\epsilon)(1-x^{\mathrm{s}}) = 1 - \epsilon(1-x^{\mathrm{s}}).$$

So with $C := \epsilon(1 - x^{\mathrm{s}}) > 0$ it follows that

$$\forall N \in \mathbb{N}\, \exists n \geq N : \int_1^\infty l_n^{\mathrm{s}}(t^{\mathrm{s}})dt^{\mathrm{s}} = 1 - \int_0^1 l_n^{\mathrm{s}}(t^{\mathrm{s}})dt^{\mathrm{s}} \geq C.$$

But this contradicts property

$$\forall n \in \mathbb{N} : \int_1^\infty l_n^{\mathrm{s}}(x^{\mathrm{s}})dx^{\mathrm{s}} = 2^{-n}.$$

Therefore it follows that

$$\forall \epsilon > 0\, \exists N \in \mathbb{N}\, \forall n \geq N : l_n^{\mathrm{s}}(x^{\mathrm{s}}) > 1 - \epsilon,$$

which means $\lim_{n\to\infty} l_n^{\mathrm{s}}(x^{\mathrm{s}}) = 1$.

Thus, the sequence weakly converges to the distribution associated with strongest aging.

It holds that

$$e_n^{\mathrm{s}}(1) = \frac{1}{l_n^{\mathrm{s}}(1)} \int_1^\infty l_n^{\mathrm{s}}(x^{\mathrm{s}}) dx^{\mathrm{s}} = 2^{2^{2n}} 2^{-n} = 2^{2^{2n}-n},$$

which means

$$\lim_{n\to\infty} S_4(l_n) = \lim_{n\to\infty} \left(1 - 2^{2^{2n}-n}\right) = -\infty.$$

Since S_4 satisfies **P5** (see below), it holds that $S_4(l_U) = 1$, so that $\lim_{n\to\infty} S_4(l_n) \not\to S_4(l_U)$, which is in contradiction to property **P4**. In fact, this also shows that even when a survival curve is close to the curve of strongest aging, S_4 might actually give values indicating arbitrarily low *negative* aging, since for every negative number c there is a N_c so that $1 - 2^{2^{2n}-n} \leq c \; \forall n \geq N_c$.

P5: Satisfied. By definition, $S_4 \leq 1$. Consider

$$l_U(x^{\mathrm{s}}) = \begin{cases} 1 & x^{\mathrm{s}} < 1, \\ 0 & x^{\mathrm{s}} \geq 1. \end{cases}$$

Since in this case,

$$e^{\mathrm{s}}(x^{\mathrm{s}}) = \begin{cases} 1 - x^{\mathrm{s}} & x^{\mathrm{s}} < 1, \\ 0 & x^{\mathrm{s}} \geq 1, \end{cases}$$

it holds that $S_4(l_U) = 1$. Conversely, assume $S_4(l) = 1$. Then $e(e_0) = 0$. Since $e(e_0) = \int_{e_0}^\infty l(x)dx/l(e_0)$, this means $l(x) = 0 \; \forall \, x \geq e_0$. This implies $l(x) = 1 \; \forall x < e_0$, because otherwise

$$e_0 = \int_0^\infty l(x)dx = \int_0^{e_0} l(x)dx < e_0.$$

It follows that $l^{\mathrm{s}} = l_U$.

Measure 5:

$$S_5(l) = 1 - \frac{e^\dagger}{e_0} = 1 - \bar{H}.$$

P1: Satisfied by definition.

P2: Satisfied because

$$\bar{H} = \bar{H}^{\mathrm{s}} = \int_0^\infty e^{\mathrm{s}}(x^{\mathrm{s}}) f^{\mathrm{s}}(x^{\mathrm{s}}) dx^{\mathrm{s}}.$$

P3: Satisfied since eq. (21) in the main text shows that S_5 is a weighted average of $-de(x)/dx$, and the derivative of e is negative if the derivative of μ is positive (and vice versa).

P4: Not satisfied. This can be shown using the same counterexample as for S_4, since for this sequence of standardized survival curves it holds that

$$\bar{H} = \bar{H}^{\mathrm{s}} = \int_0^\infty H^{\mathrm{s}}(x^{\mathrm{s}}) l^{\mathrm{s}}(x^{\mathrm{s}}) dx^{\mathrm{s}} \geq \int_1^\infty H^{\mathrm{s}}(x^{\mathrm{s}}) l^{\mathrm{s}}(x^{\mathrm{s}}) dx^{\mathrm{s}} \geq$$
$$\geq H^{\mathrm{s}}(1) \int_1^\infty l^{\mathrm{s}}(x^{\mathrm{s}}) dx^{\mathrm{s}} = \log(2) 2^{2n} 2^{-n} = \log(2) 2^n,$$

which implies

$$S_5(l_n) = 1 - \bar{H} \leq 1 - \log(2) 2^n,$$

so that

$$\lim_{n\to\infty} S_5(l_n) \nrightarrow S_5(l_U) = 1.$$

P5: Satisfied. By definition, $S_5 \le 1$. For

$$l_U(x^{\mathrm{s}}) = \begin{cases} 1 & x^{\mathrm{s}} < 1, \\ 0 & x^{\mathrm{s}} \ge 1 \end{cases}$$

it holds that $S_5(l_U) = 1$ (since $\log(1) = 0$ and, according to what we said after the introduction of S_4, $0 \cdot \log(0) := 0$). Conversely, assume $S_5(l) = 1$. Then

$$\int_0^\infty \log(l(x)) l(x) dx = 0.$$

This means that for all x: $l(x) = 1$ or $l(x) = 0$. There exists a minimal x_0 with $l(x_0) = 0$, and it follows that

$$l(x) = \begin{cases} 1 & x < x_0, \\ 0 & x \ge x_0, \end{cases}$$

and thus $x_0 = e_0$. This is exactly the distribution where everybody dies at the same age.

Measure 6:

$$S_6(l) = 1 - \frac{\sigma}{e_0} = 1 - c_v.$$

with $\sigma = \sqrt{\int_0^\infty (x - e_0)^2 f(x) dx}$. Note that to calculate the value of S_6 for non-differentiable survival functions, the expression $\sigma^2 = 2\int_0^\infty x l(x) dx - e_0^2$ can be used (it follows from applying integration by parts to $\int_0^\infty x^2 f(x) dx$ with $u(x) = x^2$ and $v'(x) = f(x)$).

P1: Satisfied by definition.

P2: Satisfied since

$$c_v = \sqrt{\int_0^\infty (x^{\mathrm{s}} - 1)^2 f^{\mathrm{s}}(x^{\mathrm{s}}) dx^{\mathrm{s}}}.$$

P3: Satisfied: As for the distinction between aging and negative aging, consider that, as mentioned, $\sigma^2 = \int_0^\infty e^2(x) f(x) dx$. It follows that for increasing μ, $e(x) \le e_0 \ \forall x$ and thus

$$\sigma^2 = \int_0^\infty e^2(x) f(x) dx \le e_0^2 \int_0^\infty f(x) dx = e_0^2,$$

so that $S_6(\mu) \ge 0$. Similarly, for decreasing μ, $e(x) \ge e_0 \ \forall x$ and thus

$$\sigma^2 = \int_0^\infty e^2(x) f(x) dx \ge e_0^2 \int_0^\infty f(x) dx = e_0^2,$$

so that $S_6(\mu) \le 0$. In the case of constant μ, clearly $\sigma = e_0$ and thus $S_6(\mu) = 0$.

P4: Not satisfied. This can be shown using the same counterexample as for S_5, since it holds that $c_v \ge \bar{H}$ and thus $S_6 \le S_5$ (see [6]).

P5: Satisfied. By definition, $S_6 \le 1$. For

$$l_U(x^{\mathrm{s}}) = \begin{cases} 1 & x^{\mathrm{s}} < 1, \\ 0 & x^{\mathrm{s}} \ge 1 \end{cases}$$

it holds that $S_6(l_U) = 1 - \sqrt{2\int_0^1 x dx - 1}/1 = 1$. Conversely, assume $S_6(l_U) = 1$. Then

$$\sigma^2 = \int_0^\infty (x - e_0)^2 f(x) dx = 0,$$

so that $f(x) \neq 0$ only if $x - e_0 = 0$, which means that all the deaths happen at the same age ($= e_0$).

Measure 7:

$$S_7(l) = \frac{2}{e_0} \int_0^\infty l^2(x)dx - 1.$$

P1: Satisfied by definition.

P2: Satisfied since

$$S_7(\mu) = 2 \int_0^\infty \left(l^{\mathrm{s}}(x^{\mathrm{s}})\right) dx^{\mathrm{s}} - 1.$$

P3: Satisfied: Apply integration by parts to $\int_0^\infty l^2(x)dx$ with $u(x) = v'(x) = l(x)$; this yields

$$\int_0^\infty l^2(x)dx = e_0 - \int_0^\infty e(x)\mu(x)l^2(x)dx.$$

But $e(x)\mu(x) = de/dx(x) + 1$, so that

$$\int_0^\infty l^2(x)dx = e_0 - \int_0^\infty (e'(x) + 1)l^2(x)dx$$
$$\Rightarrow 2 \int_0^\infty l^2(x)dx = e_0 - \int_0^\infty \frac{de}{dx}(x)l^2(x)dx,$$

and thus

$$\frac{2}{e_0} \int_0^\infty l^2(x)dx - 1 = -\frac{1}{e_0} \int_0^\infty \frac{de}{dx} l^2(x)dx,$$

which is positive for increasing μ, 0 for constant μ, and negative for decreasing μ.

P4: Satisfied. If $L(l_1^{\mathrm{s}}, l_2^{\mathrm{s}}) = \delta$, then

$$l_2^{\mathrm{s}}(x^{\mathrm{s}} - \delta) - \delta \leq l_1^{\mathrm{s}}(x^{\mathrm{s}}) \leq l_2^{\mathrm{s}}(x^{\mathrm{s}} + \delta) + \delta \quad \forall x^{\mathrm{s}}.$$

This implies

$$l_2^{\mathrm{s}}(x^{\mathrm{s}} - \delta)l_1^{\mathrm{s}}(x^{\mathrm{s}}) - \delta l_1^{\mathrm{s}}(x^{\mathrm{s}}) \leq \left(l_1^{\mathrm{s}}(x^{\mathrm{s}})\right)^2 \quad \forall x^{\mathrm{s}}$$

and thus

$$\int_0^\infty \left(l_1^{\mathrm{s}}(x^{\mathrm{s}})\right)^2 dx^{\mathrm{s}} > \int_\delta^\infty \left(l_1^{\mathrm{s}}(x^{\mathrm{s}})\right)^2 dx^{\mathrm{s}} \geq$$
$$\geq \int_\delta^\infty l_2^{\mathrm{s}}(x^{\mathrm{s}} - \delta)l_1^{\mathrm{s}}(x^{\mathrm{s}})dx^{\mathrm{s}} - \delta \int_\delta^\infty l_1^{\mathrm{s}}(x^{\mathrm{s}})dx^{\mathrm{s}} >$$
$$> \int_0^\infty l_2^{\mathrm{s}}(x^{\mathrm{s}})l_1^{\mathrm{s}}(x^{\mathrm{s}} + \delta)dx^{\mathrm{s}} - \delta.$$

It follows that

$$\int_0^\infty l_2^{\mathrm{s}}(x^{\mathrm{s}})l_1^{\mathrm{s}}(x^{\mathrm{s}} + \delta)dx^{\mathrm{s}} < \int_0^\infty \left(l_1^{\mathrm{s}}(x^{\mathrm{s}})\right)^2 dx^{\mathrm{s}} + \delta \tag{2}$$

On the other hand, $L(l_1^{\mathrm{s}}, l_2^{\mathrm{s}}) = \delta$ also implies

$$l_1^{\mathrm{s}}(x^{\mathrm{s}} - \delta) - \delta \leq l_2^{\mathrm{s}}(x^{\mathrm{s}}) \leq l_1^{\mathrm{s}}(x^{\mathrm{s}} + \delta) + \delta \quad \forall x^{\mathrm{s}},$$

so that

$$\left(l_1^{\mathrm{s}}(x^{\mathrm{s}})\right)^2 \leq l_1^{\mathrm{s}}(x^{\mathrm{s}} + \delta)l_2^{\mathrm{s}}(x^{\mathrm{s}}) + \delta l_2^{\mathrm{s}}(x^{\mathrm{s}}) \quad \forall x^{\mathrm{s}}$$

and thus

$$\int_0^\infty \left(l_2^{\mathrm{s}}(x^{\mathrm{s}})\right)^2 dx^{\mathrm{s}} \leq \int_0^\infty l_2^{\mathrm{s}}(x^{\mathrm{s}}) l_1^{\mathrm{s}}(x^{\mathrm{s}}+\delta) dx^{\mathrm{s}} + \delta.$$

It follows that

$$\int_0^\infty l_2^{\mathrm{s}}(x^{\mathrm{s}}) l_1^{\mathrm{s}}(x^{\mathrm{s}}+\delta) dx^{\mathrm{s}} > \int_0^\infty \left(l_2^{\mathrm{s}}(x^{\mathrm{s}})\right)^2 dx^{\mathrm{s}} - \delta. \tag{3}$$

(2) and (3) together imply

$$\int_0^\infty \left(l_2^{\mathrm{s}}(x^{\mathrm{s}})\right)^2 dx^{\mathrm{s}} - \int_0^\infty \left(l_1^{\mathrm{s}}(x^{\mathrm{s}})\right)^2 dx^{\mathrm{s}} < 2\delta.$$

The same reasoning with the roles of l_1^{s} and l_2^{s} reversed shows that

$$\int_0^\infty \left(l_1^{\mathrm{s}}(x^{\mathrm{s}})\right)^2 dx^{\mathrm{s}} - \int_0^\infty \left(l_2^{\mathrm{s}}(x^{\mathrm{s}})\right)^2 dx^{\mathrm{s}} < 2\delta.$$

Thus it follows that

$$|S_7(l_2) - S_7(l_1)| = 2\left|\int_0^\infty \left(l_2^{\mathrm{s}}(x^{\mathrm{s}})\right)^2 dx^{\mathrm{s}} - \int_0^\infty \left(l_1^{\mathrm{s}}(x^{\mathrm{s}})\right)^2 dx^{\mathrm{s}}\right| < 4\delta.$$

For a given $\epsilon > 0$, choosing $\delta := \frac{\epsilon}{4}$ thus implies

$$L(l_1^{\mathrm{s}}, l_2^{\mathrm{s}}) < \delta \Rightarrow |S_7(l_1) - S_7(l_2)| < \epsilon.$$

P5: Satisfied. Because $\int_0^\infty l^2(x)dx \leq \int_0^\infty l(x)dx$, clearly $S_7 \leq 1$. If l is the survival function of a distribution where everybody dies at the same age, $l^2(x) = l(x)\ \forall x$ and thus $S_7(l) = 1$. Conversely, $S_7(l) = 1$ implies $\int_0^\infty l^2(x)dx = \int_0^\infty l(x)dx$ and thus $\int_0^\infty l(x)(1-l(x))dx = 0$. This could not be the case if there existed a x with $0 < l(x) < 1$ since l is right-continuous. Thus for all x: $l(x) = 1$ or $l(x) = 0$. There exists a minimal x_0 with $l(x_0) = 0$, and it follows that

$$l(x) = \begin{cases} 1 & x < x_0, \\ 0 & x \geq x_0, \end{cases}$$

and thus $x_0 = e_0$. This is exactly the distribution where everybody dies at the same age.

References

1. Abramowitz A, Stegun I (1964) Handbook of Mathematical Functions with Formulas, Graphs, and Mathematical Tables. New York: Dover.
2. Missov TI, Lenart A (2011) Linking period and cohort life-expectancy linear increases in gompertz proportional hazards model. Demographic Research 24(19): 455-468.
3. Lenart A (2014) The moments of the gompertz distribution and maximum likelihood estimation of its parameters. Scandinavian Actuarial Journal 2014(3): 255-277.
4. Missov TI (2013) Gamma-Gompertz life expectancy at birth. Demographic Research 28: 259–270.
5. Missov TI, Lenart A (2013) Gompertz-Makeham life expectancies: Expressions and applications. Theoretical Population Biology 90: 29–35.
6. Wrycza TF (2014) Variance in age at death equals average squared remaining life expectancy at death. Demographic Research 30(50): 1405-1412.
