# Supplementary material for: Quantifying the Shape of Aging
Source: PLoS One. 2015 Mar 24;10(3):e0119163. doi: 10.1371/journal.pone.0119163 (PMC4372288; doi:10.1371/journal.pone.0119163)
Supplement: S2 Appendix — (PDF) [file pone.0119163.s002.pdf]

S2 Appendix

Here we rescale our shape measures into quantities which (in a sense that differs from measure to measure) are ratios of late-life mortality over early-life mortality. This allows for a more direct interpretation of their values.

S_1: The ratio $\mu(e_0)/\mu(0) = \mu^s(1)/\mu^s(0)$ is given by

$$\frac{\mu(e_0)}{\mu(0)} = \frac{1}{1-S_1}.$$

S_2: The ratio $\mu^\dagger/\mu(0)$ with $\mu^\dagger = \int_0^\infty \mu(x)f(x)dx$ is given by

$$\frac{\mu^\dagger}{\mu(0)} = 1 - \frac{1}{e_0\mu(0)}\log\left(1-S_2\right).$$

S_3: The ratio $\bar{\mu^s}/\bar{\mu^s}_{[0,1]}$ with $\bar{\mu}_{[0,1]} = \int_0^1 \mu^s(x^s)dx^s$ (see the discussion and caveat after the introduction of S_3) is given by

$$\frac{\bar{\mu^s}}{\bar{\mu^s}_{[0,1]}} = \frac{1}{H(e_0)} = \frac{1}{H^s(1)} = \frac{1}{1-S_3}.$$

S_4: Since $1/e(x) = \bar{\mu}(x)$ (see eq. (18) in the main text), the ratio $e_0/e(e_0)$ can be interpreted as a ratio of (average) mortality rates, and

$$\frac{\bar{\mu}(e_0)}{\bar{\mu}(0)} = \frac{e_0}{e(e_0)} = \frac{1}{1-S_4}.$$

S_5: Again, since $1/e(x) = \bar{\mu}(x)$, the ratio $e_0/e^\dagger$ can be interpreted as a ratio of (average) mortality rates, and

$$\frac{e_0}{e^\dagger} = \frac{1}{\bar{H}} = \frac{1}{1-S_5}.$$

S_6: Since $\sigma^2 = \int_0^\infty e^2(x)f(x)dx$, according to the same logic as in the case of S_4, the ratio e_0/σ can be interpreted as a ratio of (average) mortality rates, and

$$\frac{e_0}{\sigma} = \frac{1}{c_v} = \frac{1}{1-S_6}.$$

S_7: Using eq. (25) from the main text together with $\int_0^\infty l^2(x)dx = e_0(1+S_7)/2$ gives

$$\frac{\int_0^\infty -\frac{de}{dx}(x)l^2(x)dx}{\int_0^\infty l^2(x)dx} = \frac{2S_7}{1+S_7}.$$

If $w(x) := l^2(x)/\int_0^\infty l^2(x)dx$ is seen as weight function (because $\int_0^\infty w(x)dx = 1$), the left hand side is a weighted average of

$$-\frac{de(x)}{dx} = \frac{d(-\frac{1}{\bar{\mu}(x)})}{dx}.$$

Therefore, defining $\bar{\mu}^\dagger$ as the value that $\bar{\mu}$ would reach at age e_0 if $d(-\frac{1}{\bar{\mu}(x)})/dx$ was constant and equal to $\int_0^\infty -de/dx(x)w(x)dx$, i.e.,

$$\frac{1}{e_0}\left(\frac{1}{\bar{\mu}(0)} - \frac{1}{\bar{\mu}^\dagger}\right) = 1 - \frac{\bar{\mu}(0)}{\bar{\mu}^\dagger} = \int_0^\infty -\frac{de}{dx}(x)w(x)dx,$$

the value

$$\frac{\bar{\mu}^\dagger}{\bar{\mu}(0)} = \frac{1}{1-\int_0^\infty \left(-\frac{de}{dx}(x)\right) w(x)dx} = \frac{1}{1-\frac{2S_7}{1+S_7}} = \frac{1+S_7}{1-S_7}$$

then can be interpreted as ratio of (average) mortality rates.
